# Structural, Transport and Electrochemical Properties of LiFePO_4_ Substituted in Lithium and Iron Sublattices (Al, Zr, W, Mn, Co and Ni)

**DOI:** 10.3390/ma6051656

**Published:** 2013-04-29

**Authors:** Janina Molenda, Andrzej Kulka, Anna Milewska, Wojciech Zając, Konrad Świerczek

**Affiliations:** Department of Hydrogen Energy, Faculty of Energy and Fuels, AGH University of Science and Technology, al. A. Mickiewicza 30, 30-059 Krakow, Poland; E-Mails: akulka1986@o2.pl (A.K.); anna.milewska@agh.edu.pl (A.M.); wojciech.zajac@agh.edu.pl (W.Z.); xi@agh.edu.pl (K.S.)

**Keywords:** Li-ion batteries, LiFePO_4_, cathode material, Li-site substitution, Fe-site substitution, transport properties, electrochemical properties

## Abstract

LiFePO_4_ is considered to be one of the most promising cathode materials for lithium ion batteries for electric vehicle (EV) application. However, there are still a number of unsolved issues regarding the influence of Li and Fe-site substitution on the physicochemical properties of LiFePO_4_. This is a review-type article, presenting results of our group, related to the possibility of the chemical modification of phosphoolivine by introduction of cation dopants in Li and Fe sublattices. Along with a synthetic review of previous papers, a large number of new results are included. The possibility of substitution of Li^+^ by Al^3+^, Zr^4+^, W^6+^ and its influence on the physicochemical properties of LiFePO_4_ was investigated by means of XRD, SEM/EDS, electrical conductivity and Seebeck coefficient measurements. The range of solid solution formation in Li_1−3*x*_Al*_x_*FePO_4_, Li_1−4*x*_Zr*_x_*FePO_4_ and Li_1−6*x*_W*_x_*FePO_4_ materials was found to be very narrow. Transport properties of the synthesized materials were found to be rather weakly dependent on the chemical composition. The battery performance of selected olivines was tested by cyclic voltammetry (CV). In the case of LiFe_1−*y*_M*_y_*PO_4_ (M = Mn, Co and Ni), solid solution formation was observed over a large range of *y* (0 < *y* ≤ 1). An increase of electrical conductivity for the substitution level *y* = 0.25 was observed. Electrons of 3*d* metals other than iron do not contribute to the electrical properties of LiFe_1−*y*_M*_y_*PO_4_, and substitution level *y* > 0.25 leads to considerably lower values of σ. The activated character of electrical conductivity with a rather weak temperature dependence of the Seebeck coefficient suggests a small polaron-type conduction mechanism. The electrochemical properties of LiFe_1−*y*_M*_y_*PO_4_ strongly depend on the Fe substitution level.

## 1. Introduction

Due to high reversible capacity (*ca.* 170 mAh g^−1^), high chemical stability and suitable voltage *vs.* lithium anode (*ca.* 3.5 V), as well as due to lack of toxicity and the low cost of substrates, LiFePO_4_ lithium iron phosphate has drawn a vast amount of interest in terms of application in reversible lithium batteries, especially because of the possible usage in the automotive industry.

**Figure 1:**
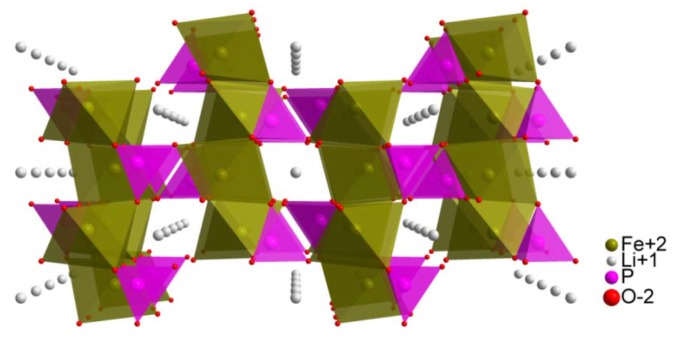
Visualization of LiFePO_4_ crystal structure, viewing along [010] direction.

The LiMPO_4_ (M = Mn, Fe, Co, Ni) group of phosphates possesses an orthorhombic *Pnma* olivine structure. They consist of edge-sharing layers of disordered MO_6_ octahedra, which are connected by PO_4_ tetrahedra. The layers are parallel to the *bc* plane. Octahedral 4*a* positions are occupied by Li^+^ cations, which form one-dimensional chains along the *b* and *c* axis (Figure 1).

The limiting factor for the performance of LiMPO_4_-based batteries is related to the material’s low electrical conductivity, both ionic and electronic. The electronic component of the conductivity is due to magnetic, hole-type polarons [1]. The spin of the hole, which in LiFePO_4_ is related to the Fe^3+^ cation exhibiting (t_g_↑)^3^(e_g_↑)^2^ electronic configuration, is ferromagnetically coupled to surrounding Fe^2+^ (t_g_↑)^3^(e_g_↑)^2^t_g_↓ cations via a double-exchange mechanism. Because of this, moving electrons, which are followed by polaron-type distortion, also carry a cloud of spin polarized charge [2]. Existence of Fe^3+^ cations is due to the presence of lithium vacancies, which may appear during the synthesis procedure as a result of evaporation of lithium and/or reaction with moisture.

The ionic component of electrical conductivity is related to the mobility of Li^+^ ions, which is preferential along [010] direction. Theoretical calculations by Islam *et al.* [3] show that in the olivine structure there are three types of diffusion paths for lithium cations: the mentioned one along [010] direction, which is characterized by 0.55 eV activation energy E_a_; one along [001] direction with activation energy equal 2.89 eV and in [101] direction with E_a_ = 3.36 eV (Figure 1). Transfer of lithium ions between the pathways is impossible due to the high value of the energy barrier equal to 2.2 eV and the long distance >4.5 Å. As can be deduced from E_a_ values, [010] direction is favored, and can be considered as a 1-dimensional pathway for Li^+^ migration, which occurs during charge and discharge of the lithium battery. Such an anisotropic structure with one-dimensional diffusion ways results in the conclusion that a plate-like grain shape with a well-developed (010) surface should exhibit enhanced performance while working in Li-ion cells. 

Literature data regarding transference numbers of electronic and ionic conductivity in phosphoolivine are not consistent [4,5]. However, on the basis of results presented by Maier [6] it is plausible that the electronic component should be dominant. The observed, total conductivity of LiFePO_4_ at room temperature is of the order of 10^−9^ Scm^−1^, with activation energy equal to about 0.65 eV [2,7].

There are literature works suggesting relationship between electronic and ionic conductivity in LiFePO_4_ [8]. It was observed that faster transport of the small polaron appears together with disordering of lithium cations, which hints at their mutual connection. Therefore high electrical resistivity may be considered responsible for the low values of lithium diffusion coefficient D. Values of D reported in the literature fall over quite a wide range from 10^−16^ to 10^−11^ cm^2^ s^−1^ and their poor reproducibility indicates the strong influence of the material’s preparation method [9,10,11,12,13].

Delithiation of LiFePO_4_ can be generally described as a two phase-type mechanism: LiFePO_4_ − *x*Li^+^ − *x*e^−^ ↔ (1 − *x*)LiFePO_4_ + *x*FePO_4_. There are several simplified models explaining the nature of this reaction, among them, the so-called *shrinking core* (*core-shell*) [14], *mosaic* [15], *new core-shell* [16] and *domino-cascade* [17] models are of interest. Moreover, extensive research into the theoretical description of chemical kinetics performed by Bazant’s group [18,19,20] allowed authors to describe quantitatively the observed behavior of Li*_x_*FePO_4_ during the lithium intercalation/deintercalation process without *ad hoc* assumptions made for simplified models [14,15,16,17].

There are only a few ways to overcome issues related to low conductivity of phosphoolivine. Among them: addition of the appropriate amount of carbonaceous additive, modification of the synthesis procedure, which yields flat nanocrystallites with a well-developed (010) surface, and substitution of iron by aliovalent cations are the most common [21,22]. While there are many works proving the possibility of substitution of iron by other 3*d* metals [23,24], results obtained for Li-site substituted materials are not decisive and this issue still seems to be an open question [25,26,27,28,29,30,31,32,33,34,35,36]. It is worth noting that for quite a long time research papers showing metallic-like properties of pure and Li-site substituted LiFePO_4_ were being published [25,27].These experimental works were followed and supported by theoretical calculations [35]. However, finally these results were questioned, and metallic-like behavior of the samples was shown to originate from metallic phosphides and carbo-phosphides, which were present on the surface of phosphoolivine grains [37,38]. Therefore, there is an ongoing discussion about possibility, range of substitution and influence of other cations with higher valence in lithium sublattices on the physicochemical properties of LiFePO_4_. 

As already mentioned, there is a vast amount of papers devoted to Fe-site substituted materials. The conducted research concerns both, end-members with LiMnPO_4_ [39], LiNiPO_4_ [40], and LiCoPO_4_ [41] compositions, as well as partially substituted LiFe_1−*x*_M*_x_*PO_4_ compounds [42,43]. The published data shows mostly only electrochemical properties of the samples. Additionally, the available data shows no significant influence of a type of 3*d* metal on the values of lithium diffusion coefficient in all studied LiMPO_4_ (M = Fe, Ni, Co) compositions [44]. Many authors suggest that the energy barrier for movement of M^3+^ polarons in the case of LiMnPO_4_ and LiCoPO_4_ is higher than for LiFePO_4_, which leads to worse transport properties of these materials [45,46]. Looking at the available literature data one notices a lack of systematic studies on the transport properties of 3*d* metal substituted compounds. 

In this review-type paper, systematic studies, conducted by our research group are presented. Complex measurements of structural, transport and electrochemical properties of phosphoolivines substituted in Li sublattices by Al^3+^, Zr^4+^ and W^6+^ cations, as well as Fe-site substituted materials LiFe_1−*x*_M*_x_*PO_4_ (M = Mn, Co and Ni) are provided. All materials were synthesized by a standard ceramic high-temperature method. Crystal structure was investigated by means of XRD, SEM-EDX and TEM techniques. Electrical conductivity was examined by DC 4-probe as well as impedance a spectroscopy method, Seebeck coefficient was evaluated from the slope of the dependence of thermoelectric voltage *vs.* applied thermal gradient. Electrochemical studies were performed in Li/Li^+^/phospholivine type cells. More details about synthesis and experimental methods can be found in references [7,47,48,49].

## 2. Results and Discussion

### 2.1. Phosphoolivines Substituted in Li Sublattice by Al^3+^, Zr^4+^ and W^6+^ Cations

#### 2.1.1. Structural Properties

**Figure 2:**
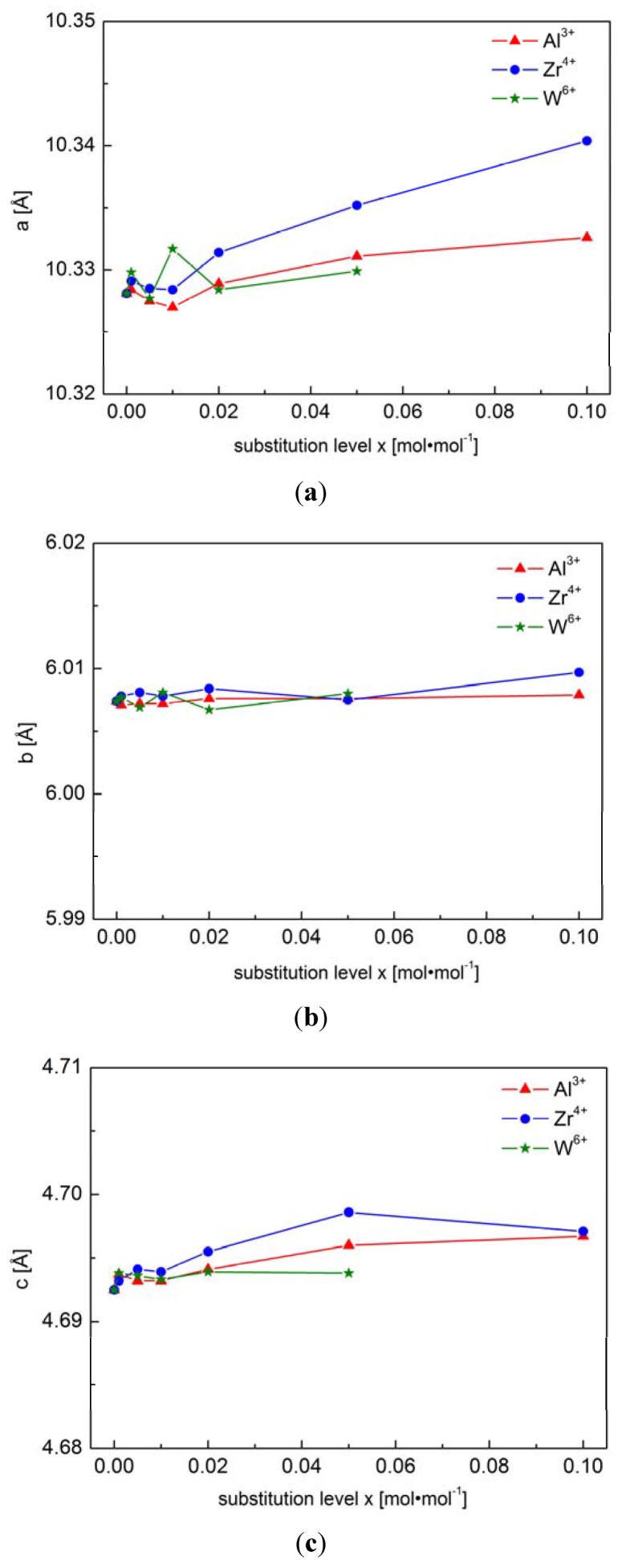
Unit cell parameters *a*, *b* and *c* for Li_1−3*x*_Al*_x_*FePO_4_, Li_1−4*x*_Zr*_x_*FePO_4_ and Li_1−6*x*_W*_x_*FePO_4_ materials [47].

Figure 2 shows unit cell parameters of Al^3+^, Zr^4+^ and W^6+^-substituted phosphoolivines as a function of concentration of introduced cations. No simple, linear dependence can be observed between level of substitution and unit cell parameters. Additionally, XRD-based phase analysis indicates a limited range of “solubility”. In the case of Zr^4+^-substituted compounds, the presence of additional Fe_3_(PO_4_)_2_ and Li_2_ZrP_2_O_8_ phases was confirmed for concentrations of *x* ≥ 0.02 mol mol^−1^. For W^6+^-substituted materials, Fe_2_P_2_O_7_ and FeWO_4_ phases were identified for doping level *x* ≥ 0.01 mol mol^−1^. Interestingly, in the case of the whole Li_1−3*x*_Al*_x_*FePO_4_ series, apart from the main orthorhombic *Pnma* phase, no secondary phases were visible on XRD patterns. However, the XRD data showed low signal/noise ratio, which further decreases with increasing Al^3+^ concentration, suggesting poor crystallinity of the samples and the possibility of formation of amorphous, glass-like phases. High noise level may also obscure the presence of peaks originating from secondary phases.

**Figure 3:**
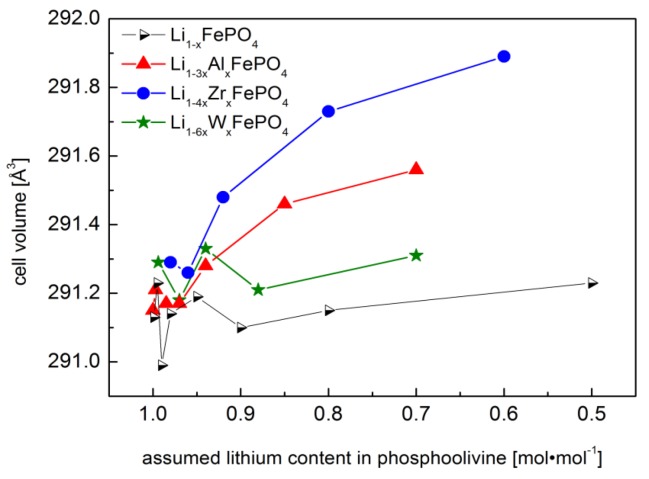
Effect of dopant concentration on the unit cell volume for pure and substituted phosphoolivines [47].

In the course of the studies, an experiment was performed, which could further explain the influence of concentration of lithium vacancies on the unit cell volume *V* of non-substituted Li_1−*x*_FePO_4_ and could help in the separation of effects induced by aliovalent substitution at the Li-site together with lithium deficiency. XRD results obtained for materials from the Li_1−*x*_FePO_4_ series, together with data for the Al^3+^, Zr^4+^ and W^6+^-substituted compounds are collated in Figure 3, in which the measured values of unit cell volume are presented as a function of lithium content. 

A nonlinear relationship between the unit cell volume and the lithium vacancy concentration in pure Li_1−*x*_FePO_4_ phosphoolivine is observed. In the case of Al^3+^ or Zr^4+^-substituted materials, an increase of unit cell volume can be noticed at higher concentration of dopant metals. However, it is present outside of the solid solution range for Li_1−4*x*_Zr*_x_*FePO_4_, while in the case of Li_1−3*x*_Al*_x_*FePO_4_ it concerns poorly crystallized samples. In the case of W^6+^-substituted materials no similar effect was observed. Taking into account ionic radii only (according to Shannon [50], octahedral coordination: Li^+^—0.76 Å, Al^3+^—0.535 Å, Zr^4+^—0.72 Å and W^6+^—0.6 Å), one may assume that the observed results do not follow the anticipated trends for changes of unit cell volume of substituted materials. Therefore, other effects must be taken into account, *i.e.*, the presence of additional lithium vacancies, and the presence of secondary phases, whose level does not permit detection during standard XRD experiments. Another reason may be related to the difference of valence and electronegativity of the dopant metals. Introduction of *d*-type cations into octahedral lithium sites may cause stronger Coulomb repulsion between cations, and electrons originating from ligands, which, in turn, may generate internal stress.

The investigations conducted on Li-site substituted phosphoolivine proved that the range of the formation of solid solution is strongly dependent on the valence and radii of the introduced metal and is limited. The solid solution formation range can be ordered as follows: *x*_maxW6+_ < *x*_maxZr4+_ < *x*_maxAl3+_, with the real values being less than, respectively: *x*_maxW6+_ < 0.01, *x*_maxZr4+_ < 0.02, *x*_maxAl3+_ < 0.1.

#### 2.1.2. Morphology of LiFePO_4_ and Li_0.97_Al_0.01_FePO_4_ Powders

**Figure 4:**
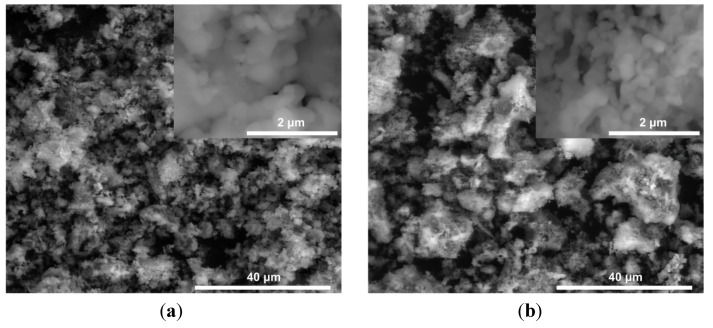
SEM microphotographs of (**a**) LiFePO_4_ and (**b**) Li_0.97_Al_0.01_FePO_4_ powders.

**Figure 5:**
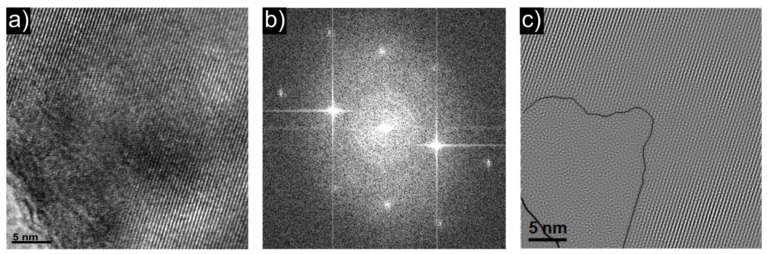
(**a**) HRTEM micrograph; (**b**) electron diffraction picture; and (**c**) HRTEM-based, FFT-analyzed picture of the structure of Li_0.97_Al_0.01_FePO_4_ material.

Figure 4 and Figure 5 show exemplary results of microstructural analysis of LiFePO_4_ and Li_0.97_Al_0.01_FePO_4_ powders, conducted by SEM and TEM techniques. Studies were supported by additional EDX measurements. As can be seen, both powders consist of irregular, few-hundred-nanometer size crystallites, which are grouped into irregular aggregates and agglomerates. Higher magnification (inlets) allows the visualization of well-sintered crystallites with the possible presence of a glass-like phase.

EDX spectroscopy analysis of a Li_0.97_Al_0.01_FePO_4_ sample indicated homogenous distribution of Al within resolution of the method (several μm^2^). Additionally, the presence of oxygen-depleted grains was confirmed, which indicates the possibility of the formation of phosphides. Further measurements on a TEM Tecnai G2 F20 microscope, equipped with a high-resolution EDX probe, enabled the confirmation of homogenous distribution of Al also in nano-scale. These measurements also confirmed the presence of regions with lower-than-expected concentration of oxygen. Additionally, neutron diffraction data (not shown in this paper, A. Braun, private communication) indicate increasing oxygen deficiency with increasing dopant amount. Interestingly, HRTEM measurements conducted on a Li_0.97_Al_0.01_FePO_4_ sample showed the presence of an amorphous phase within single grains of the material (Figure 5). These regions, present in virtually all particles, have almost the same chemical composition as the surrounding crystalline phase.

The results presented above allows us to draw conclusion that while actual substitution in the Li-sublattice by Al^3+^ in phosphoolivine is likely, it also causes formation of a significant amount of amorphous phase. The solid solution range *x* is definitely lower than 0.1 mol·mol^−1^, but precise determination of the limit is not possible. 

#### 2.1.3. Transport Properties

**Figure 6:**
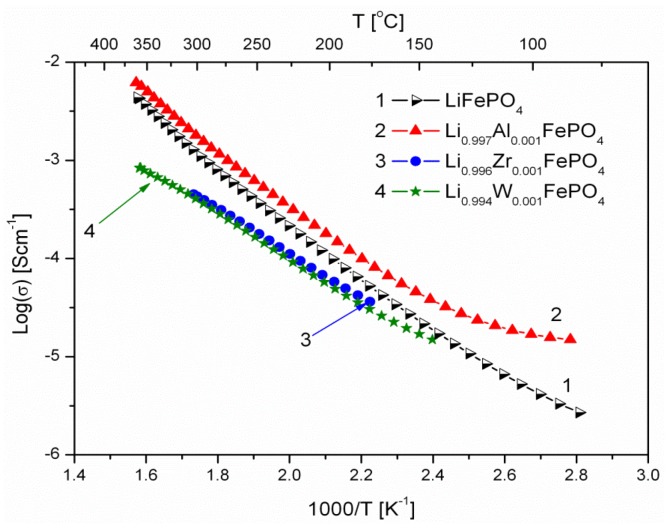
Temperature dependence of electrical conductivity of Li-site substituted phosphoolivines with *x* = 0.001 mol mol^−1^.

**Figure 7:**
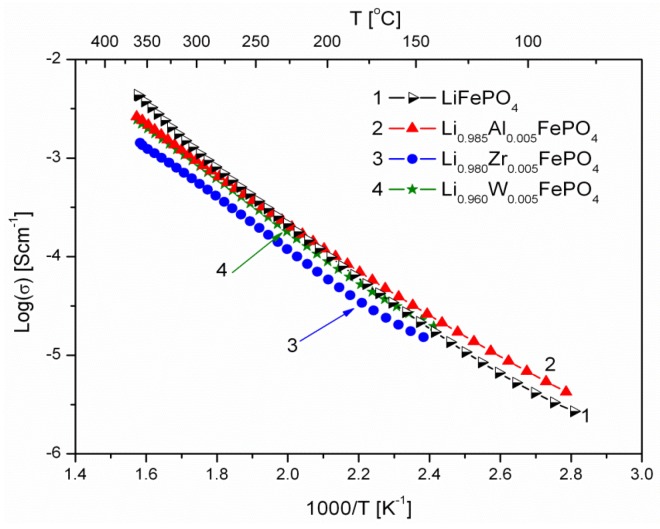
Temperature dependence of electrical conductivity of Li-site substituted phosphoolivines with *x* = 0.005 mol mol^−1^.

**Figure 8:**
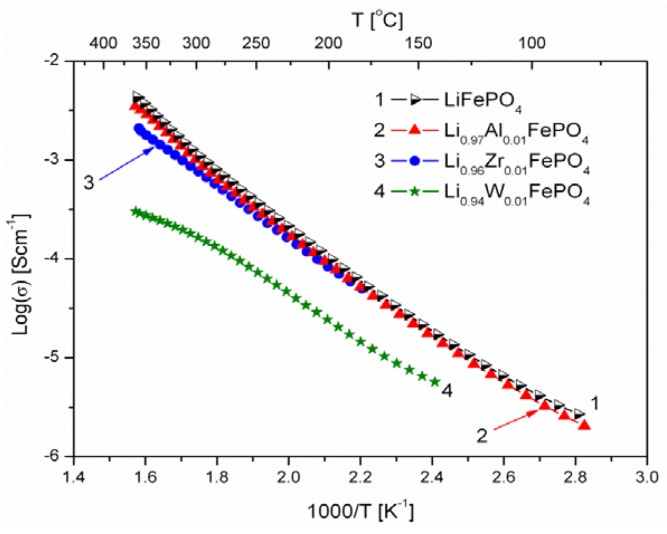
Temperature dependence of electrical conductivity of Li-site substituted phosphoolivines with *x* = 0.01 mol mol^−1^.

**Figure 9:**
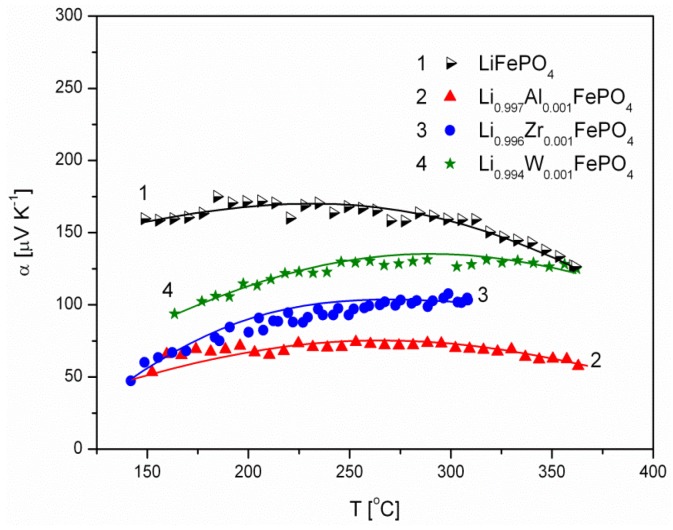
Temperature dependence of thermoelectric power of Li-site substituted phosphoolivines with *x* = 0.001 mol mol^−1^.

**Figure 10:**
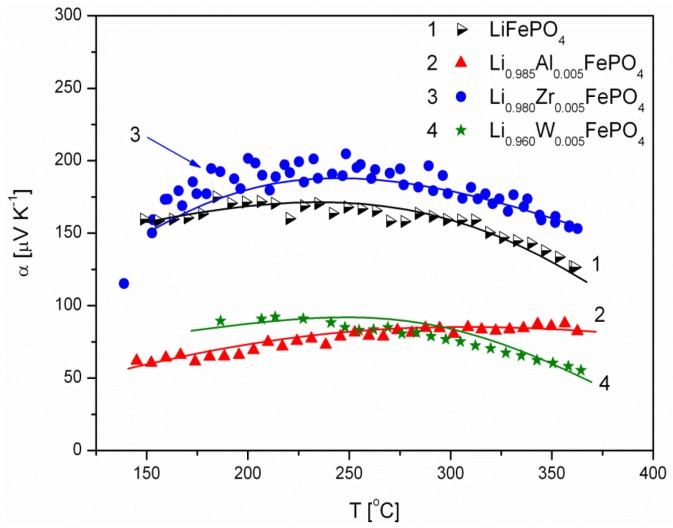
Temperature dependence of thermoelectric power of Li-site substituted phosphoolivines with *x* = 0.005 mol mol^−1^.

**Figure 11:**
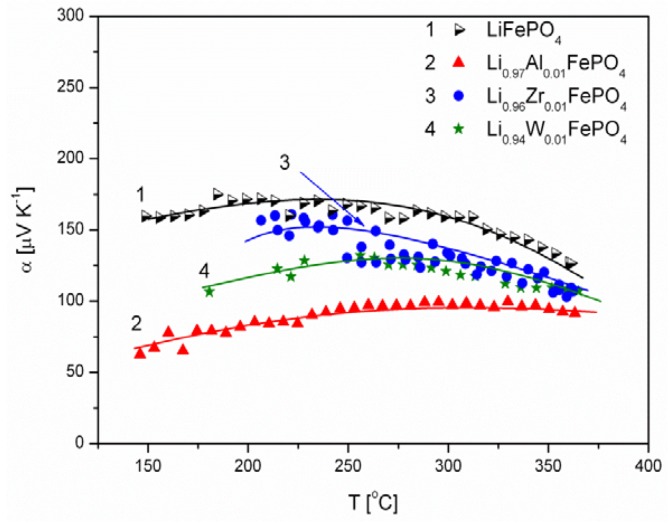
Temperature dependence of thermoelectric power of Li-site substituted phosphoolivines with *x* = 0.01 mol mol^−1^.

Figure 6, Figure 7, Figure 8, Figure 9, Figure 10 and Figure 11 show the results of the measurements of total electrical conductivity *σ* and thermoelectric power *α* of phosphoolivines substituted in the Li-sublattice by Al^3+^, Zr^4+^ and W^6+^ cations, respectively, data taken from [47]. All the results are shown for samples within the assumed range of formation of solid solution (*x*_W6+_ < 0.01, *x*_Zr4+_ < 0.02, *x*_Al3+_ < 0.1). For comparison, data for pristine LiFePO_4_ is also given. Data for Li_1−3*x*_Al*_x_*FePO_4_ is taken from [48].

As indicated by results shown in Figure 6, Figure 7 and Figure 8, the temperature dependence of electrical conductivity for all studied samples exhibits activated character. In the case of Al^3+^-substituted materials, no significant effect of aluminum on σ values can be seen, apart from a somewhat unexpected behavior for Li_0.997_Al_0.001_FePO_4_ at lower temperatures. Assuming Li substitution by Zr and W causes a decrease of electrical conductivity of the materials, however this effect may also originate from the secondary phases, whose presence is evident at higher concentration levels. Estimated values of the activation energy of electrical conductivity E_a_ are in the 0.44–0.6 eV range, and do not differ significantly from pristine LiFePO_4_, for which a similar range of E_a_ values was reported, dependent on the synthesis method.

The positive sign of thermoelectric power (Figure 9, Figure 10 and Figure 11) for Al^3+^, Zr^4+^ and W^6+^-substituted materials indicates positive charge carries (holes) as dominant. This is characteristic for phosphoolivines obtained by the high-temperature method, and can be related to the evaporation of lithium during the procedure over the 650–750 °C temperature range, formation of lithium vacancies (V′_Li_) and charge compensation by creation of holes (Fe˙_Fe_). Lower values of α, measured for substituted phosphoolivines in comparison to LiFePO_4_, may be related to the presence of phosphides on the phosphoolivine grains.

The authors’ studies on synthesis methods show that depending on the conditions of preparation of the substituted materials (Li_1−3*x*_Al*_x_*FePO_4_, Li_1−4*x*_Zr*_x_*FePO_4_ and Li_1−6*x*_W*_x_*FePO_4_, 0.001 < *x* < 0.1), *i.e.*, final temperature and heating rate, Ar flow rate, its purity and type of substrates, the observed values of electrical conductivity may vary as much as in the range 10^−9^–10^−4^ S cm^−1^ at room temperature. Also, the activation energy E_a_ changes from 0.7 eV down to as low as 0.02 eV. However, critical analysis of the observed electrical properties, which were measured for a large group of substituted phosphoolivines as a function of concentration and type of introduced cation, unambiguously falsifies the hypothesis that high values of the order of 10^−4^ S cm^−1^ at room temperature are related to a bulk conductivity. This is because of the following fundamental objections:

- concentration and type of dopant have no significant influence on the values of electrical conductivity,
- no critical concentration was observed, for which an effective conduction band could be created as an in Mott-type metal-insulator transition,
- inhomogeneous distribution of dopant elements may also lead to high conductivity values,
- the delithiation process proceeds as a two-phase type reaction, the same as in the case of insulating LiFePO_4_ (see the following section).

The analysis of synthesis procedure conditions for phosphoolivine materials and the type of selected substrates lead to the conclusion that enhanced conductivity appears as a result of formation of a conduction path, due to precipitation of carbon (from organic precursors) and/or formation of a thin conduction path consisting of metallic-type iron phosphides (Fe_2_P), which appear in reductive synthesis conditions. Traces of such iron phosphides, estimated as 3–4 wt %, were detected by the surface-sensitive CEMS Moessbauer technique [49] and TEM studies [37]. It can be therefore stated [38] that conductive phosphoolivine is in fact a composite material comprising an insulating LiFePO_4_ core and a non-continuous layer of conducting Fe_2_P and carbon on the surface of the grains. Iron phosphides and carbon precipitates form a conducting percolation path, which is manifested macroscopically in a form of enhanced, like metallic conductivity. Using the following substrates: FeC_2_O_4_·H_2_O, NH_4_H_2_PO_4_ and Li_2_CO_3_, in the course of the synthesis, and with oxygen partial pressure <10^−5^ atm, there are several internal reducing agents: pyrophoric Fe (the strongest one), iron carbonyls Fe*_x_*(CO)*_y_*, as well as C, CO and NH_3_. All of them together lead to partial reduction of LiFePO_4_ to Fe_2_P. Furthermore, an assumed lithium deficiency (in Li_1−*x*_FePO_4_), causes the appearance of the Fe^2+^/Fe^3+^ redox pair, which may further catalyze reduction of LiFePO_4_ to Fe_2_P.

Impedance spectroscopy measurements of the electrical conductivity of pristine LiFePO_4_, performed over the 300–700 K temperature range [7], allowed the determination of an ionic and an electronic component of electrical conductivity. Both components are comparable and are of the order of 10^−9^ S cm^−1^ at room temperature. Also, the activation energies of both components are comparable and are equal to 0.66 eV and 0.63 eV for electronic and ionic conductivity, respectively. It is generally acknowledged that iron ions in the octahedral position in Li_1−*x*_FePO_4_ possess high-spin configuration: t^3^_t2g_(↑)e^2^_g_(↑)t_t2g_(↓) for Fe^2+^ and t^3^_t2g_(↑)e^2^_g_(↑) for Fe^3+^. The energy gap E_g_ in LiFePO_4_ was determined by theoretical [51] and experimental [2] methods and is of the order of 3.8 eV. Therefore, the observed activation energy of the electronic component of electrical conductivity (0.66 eV) may be explained as the migration energy of Fe˙_Fe_-V′_Li_ pairs, which form so called magnetic polarons. Studies of magnetic properties [2] confirmed the presence of such magnetic polarons and their concentration was estimated as being equal to 0.2–0.3 mol %.

As was confirmed by our studies, the effect of non-symmetrical cation mixing, which leads to the formation of complex Fe˙_Li_-V′_Li_ defects with Fe^2+^ions present in lithium sites, results in the increase of the activation energy of lithium migration, even up to 0.8 eV.

Analysis of the effect of Al^3+^, Zr^4+^ and W^6+^ cations on the transport properties of LiFePO_4_ leads to the conclusion that it manifests itself mainly through an increase in concentration of lithium vacancies, due to charge compensation requirements and associated changes of unit cell parameters. Therefore, it affects ionic conductivity much stronger, which can be observed as a decrease of activation energy of ionic conductivity from 0.7 eV down to 0.48–0.45 eV. This is mainly associated with an increase of unit cell parameters and volume, as well as with an increase in concentration of lithium vacancies.

#### 2.1.4. Electrochemical Properties

**Figure 12:**
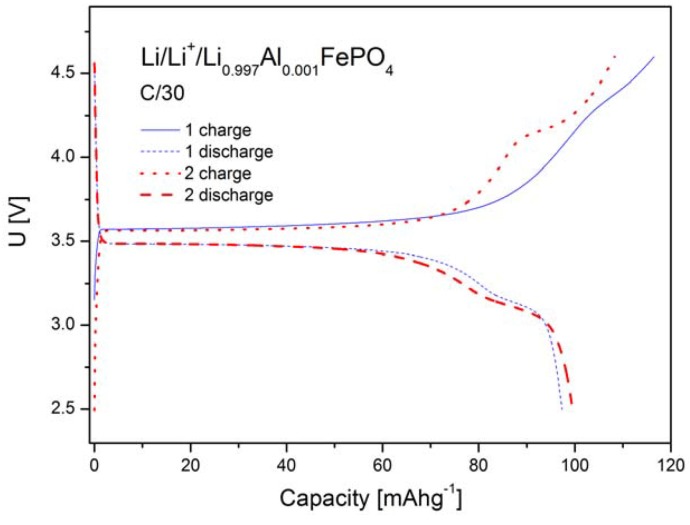
Charge and discharge curves (two initial cycles) for Li/Li^+^/Li_0.997_Al_0.001_FePO_4_ cell.

**Figure 13:**
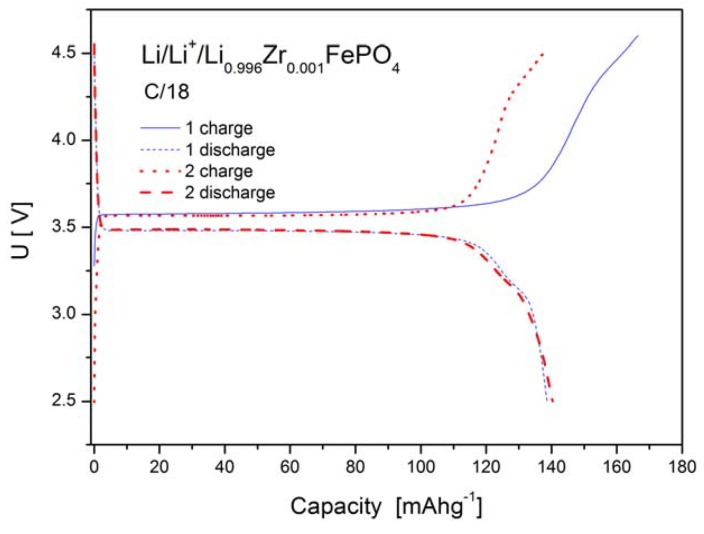
Charge and discharge curves (two initial cycles) for Li/Li^+^/Li_0.996_Zr_0.001_FePO_4_ cell.

**Figure 14:**
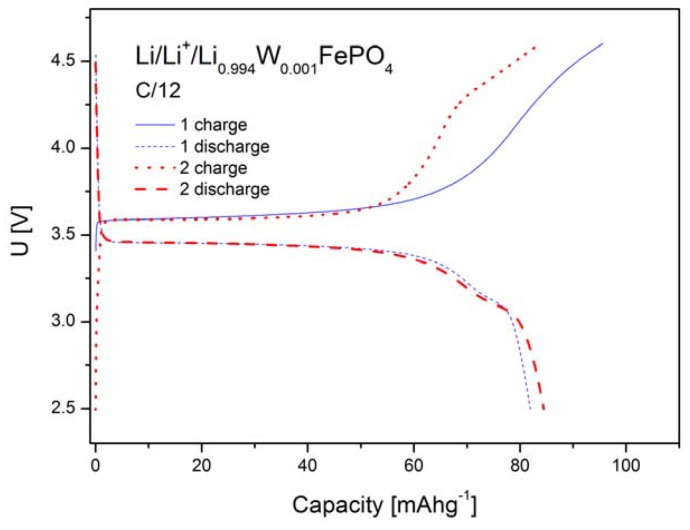
Charge and discharge curves (two initial cycles) for Li/Li^+^/Li_0.994_W_0.001_FePO_4_ cell.

Figure 12, Figure 13 and Figure 14 show charge and discharge curves of selected lithium cells, in which studied, Li-site substituted phosphoolivines were used as the cathode material.

As can be seen in Figure 12, Figure 13 and Figure 14, charge and discharge processes present plateaux, of which voltage *versus* lithium anode is equal to about 3.6 V and slightly below 3.5 V, respectively. During the charging of the cell, lithium cations, together with an equal amount of electrons from t_t2g_(↓) level, are taken out from the cathode material, which causes oxidation of Fe^2+^ to Fe^3+^. The reverse process takes place during the cell’s discharge. The plateau-type character of the curves indicates a two-phase type mechanism of the cathode process and is in general agreement with predictions of state-of-the-art theoretical models [18,19,20] for low density current regime and in the morphology of investigated powders Simplifying, the following equation can describe the intercalation/deintercalation mechanism of phosphoolivine: 

$$LiFePO_{\;\;4}-xLi^{\;+}-xe^{-}\overset{charge}{\underset{discharge}{↔}}(1-x)Li_{\;1}FePO_{\;\;4}+xFePO_{\;\;4}$$

**Figure 15:**
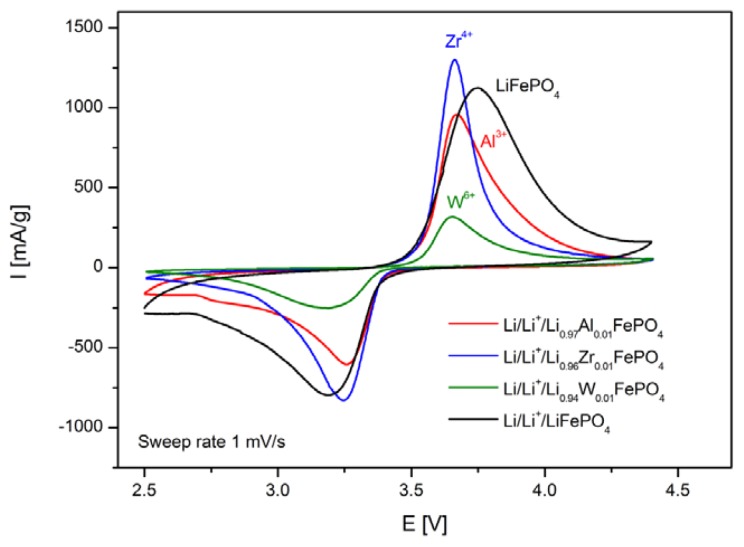
Cyclic voltammograms recorded for lithium cells with Li_0.97_Al_0.01_FePO_4_, Li_0.96_Zr_0.01_FePO_4_, Li_0.94_W_0.01_FePO_4_ and LiFePO_4_ cathode materials [47].

Figure 15 shows results of cyclic voltammetry studies obtained for selected cells based on LiFePO_4_, Li_0.97_Al_0.01_FePO_4_, Li_0.96_Zr_0.01_FePO_4_ and Li_0.94_W_0.01_FePO_4_ cathode materials. For all phosphoolivine cathode materials only a single redox peak is present. It can be observed that cathodic peaks are significantly higher, compared to anodic ones, which effect may be connected to different kinetics of delithiation and lithiation. Also, the shape and size of redox peaks depend on the dopant type. The highest and most narrow peaks can be seen on voltammetric curves recorded for Li_0.96_Zr_0.01_FePO_4_-based cells, while the smallest occurs for Li_0.94_W_0.01_FePO_4_-based cathode material. This is in agreement with charge/discharge curves and the corresponding capacity of the cathode materials, presented in Figure 12, Figure 13 and Figure 14. In summary, Zr^4+^ and Al^3+^doped materials exhibit similar properties to undoped LiFePO_4_, while the W^6+^ doped compound behaves much worse.

**Figure 16:**
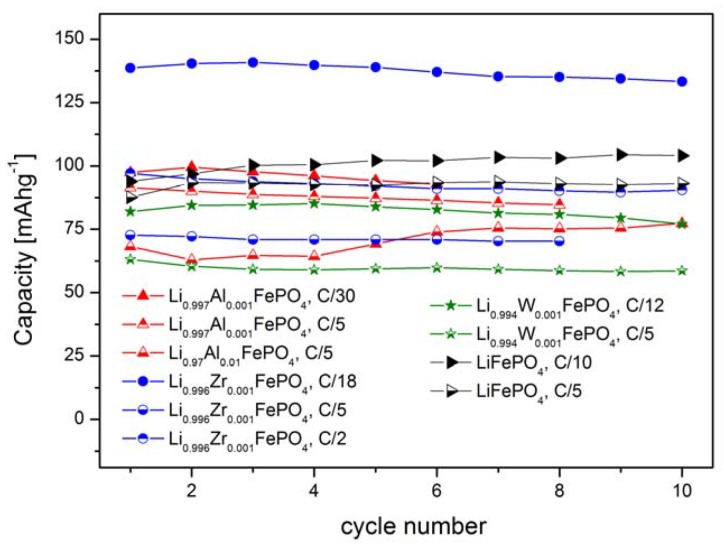
Reversible capacity of studied lithium cells with Li_0.997_Al_0.001_FePO_4_, Li_0.996_Zr_0.001_FePO_4_, Li_0.994_W_0.001_FePO_4_ and Li_0.97_Al_0.01_FePO_4_ cathode materials as a function of cycle number. Data obtained for different charge/discharge rates.

Such an effect of substitution can be further confirmed by data regarding reversible capacities of cells with Li_0.997_Al_0.001_FePO_4_, Li_0.996_Zr_0.001_FePO_4_, Li_0.994_W_0.001_FePO_4_ and Li_0.97_Al_0.01_FePO_4_-based cathodes, which were obtained for different charge/discharge rates (Figure 16). The best results were obtained for Li_0.996_Zr_0.001_FePO_4_ cathode material. 

### 2.2. LiFePO_4_ Substituted by Mn^2+^, Co^2+^, Ni^2+^ and Cu^2+^ Cations in the Iron Sublattice 

Three series of LiFe_1−*y*_M*_y_*PO_4_ materials were studied, in which the amount of substitution y of metal M = Mn^2+^, Co^2+^ and Ni^2+^ was equal to 0, 0.25, 0.5, 0.75 and 1 mol mol^−1^. An attempt to synthesize LiFe_1−*y*_Cu*_y_*PO_4_ phosphoolivines for *y* = 0.01, 0.02 and 0.05 mol mol^−1^ was also conducted. In all cases a high-temperature solid state reaction method was applied.

#### 2.2.1. Structural Properties

**Figure 17:**
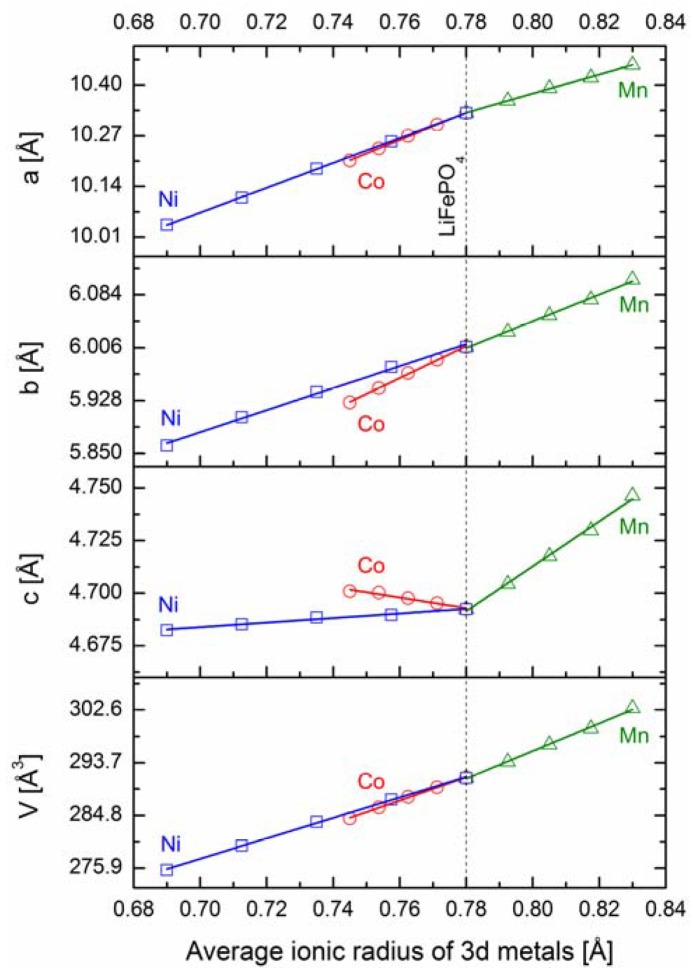
Unit cell parameters and volumes for LiFe_1−*y*_M*_y_*PO_4_ (M = Mn, Co, Ni) as a function of average ionic radius of Fe_1−*y*_M*_y_*.

Figure 17 shows the dependence of unit cell parameters *a*, *b* and *c*, as well as the unit cell volume *V* of studied LiFe_1−*y*_M*_y_*PO_4_ (M = Mn, Co, Ni) materials as a function of average ionic radius of 3*d* metal cations Fe_1−*y*_M*_y_*. The average radius was calculated as the weighted average using Shannon’s ionic radii data for octahedral coordination, assuming +2 valence and high-spin state of 3*d* metals [50]. 

According to Vegard’s law, the observed linear dependence indicates formation of solid solution in the whole range of studied compositions. In all cases, however, *a* and *b* parameters behave in a different way to parameter *c*. This can be justified taking into account two features of phosphoolivine-type crystal structure: stiffness of P–O bonds and placement of PO_4_ tetrahedra (Figure 1). Along the *a*-axis this structure can be considered as a layered one, in which layers of MO_6_ octahedra are connected by PO_4_ polyanions and lithium cations [1]. Therefore, the thickness of such layers is not dependent on the size of PO_4_ tetrahedra, which affects only the interlayer distance. As a result, the thickness of these layers and the corresponding unit cell parameter *a* will change linearly with changes of the average radius of 3*d* metal cations. However, PO_4_ tetrahedra may affect the size of MO_6_ octahedra along the *b* and *c* direction. This is caused by the fact that these tetrahedra and octahedra have common edges (along *b* axis) and vertices (along *c* axis). Substitution of iron by 3*d* metals which are too small would cause an unfavorably high decrease of the P–O bong length, which in pristine LiFePO_4_ is already relatively short, compared to typical values of tetrahedrally coordinated P^5+^ cation by O^2−^ anions. This effect seems to explain the observed asymmetry between compressive and tensile stresses in the substituted materials. In the case of phosphoolivine substituted by Ni, only a small decrease of the P–O bond length (decrease of parameter *c*) was observed, while in the case of LiFe_1−*y*_Mn*_y_*PO_4_, a substantial increase of unit parameter *c* indicates relaxation of the P–O bond length. For LiFe_1−*y*_Co*_y_*PO_4_ materials, parameter *c* increases unexpectedly, with the increase of cobalt content, as Co^2+^ cations are smaller, compared to Fe^2+^. This suggests the existence of some additional interactions. 

Substitution of iron by Cu^2+^ cations is not possible over a wide range of chemical composition. For samples, in which the amount of copper did not exceed 5 mol % no additional phases were detected in the XRD patterns, however, the lack of systematic changes of unit cell parameters suggests that the range of formation of solid solutions is very narrow and does not exceed 1–2 mol %.

#### 2.2.2. Transport Properties

**Figure 18:**
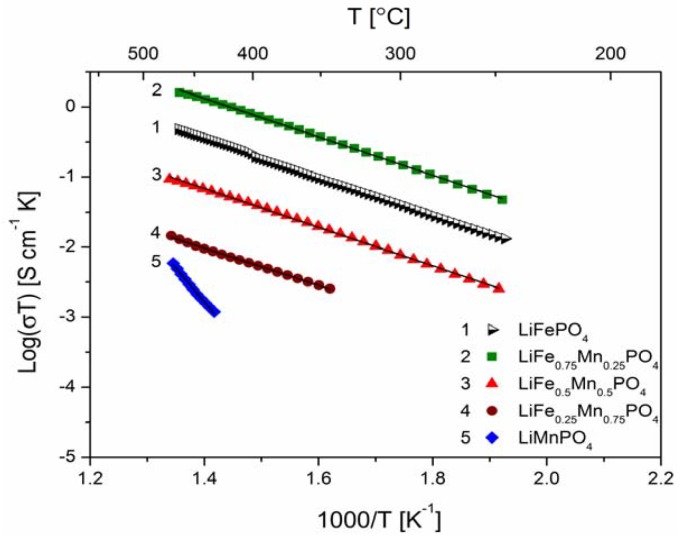
Temperature dependence of electrical conductivity of LiFe_1−*y*_Mn*_y_*PO_4_.

**Figure 19:**
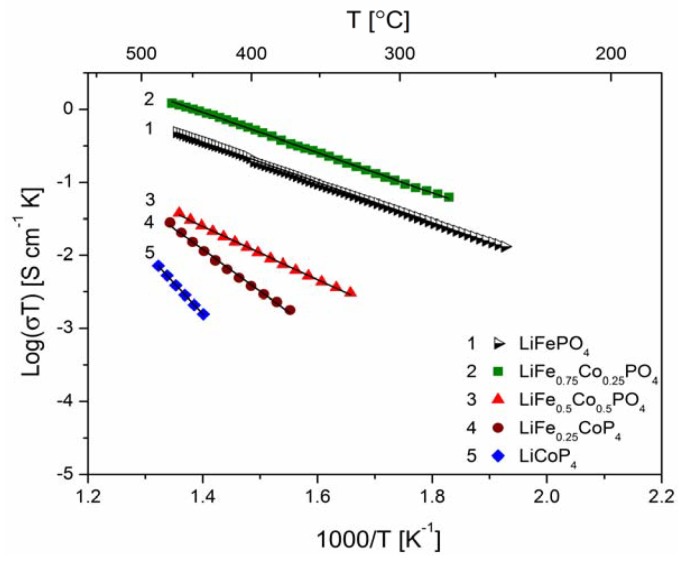
Temperature dependence of electrical conductivity of LiFe_1−*y*_Co*_y_*PO_4_.

**Figure 20:**
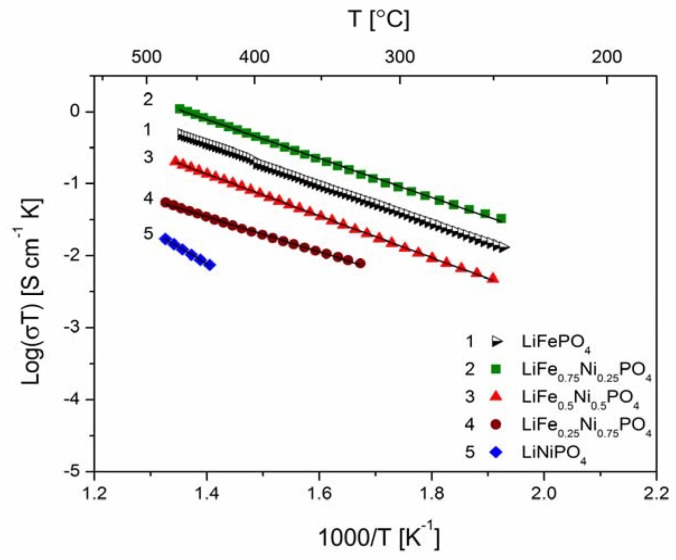
Temperature dependence of electrical conductivity of LiFe_1−*y*_Ni*_y_*PO_4_.

**Figure 21:**
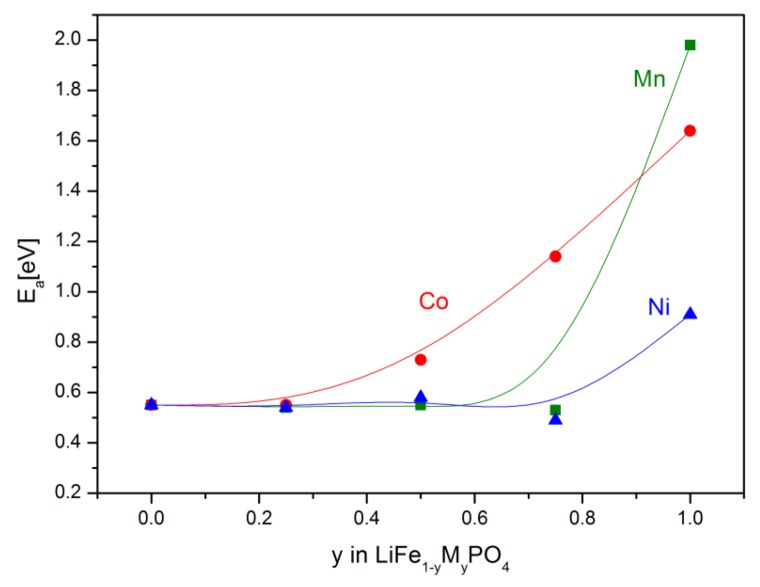
Chemical composition dependence of activation energy of electrical conductivity E_a_ for LiFe_1−*y*_M*_y_*PO_4_ (M = Mn, Co, Ni) olivines.

**Figure 22:**
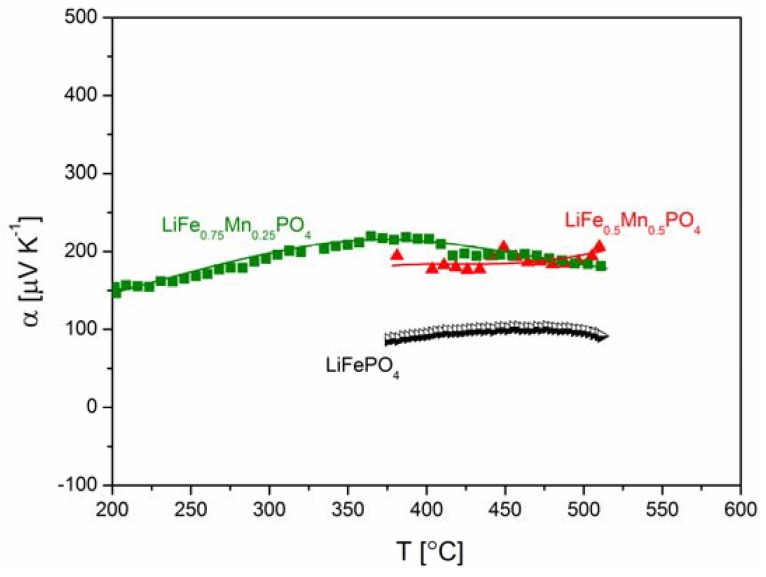
Temperature dependence of thermoelectric power of Li_1−*y*_Mn*_y_*PO_4_.

**Figure 23:**
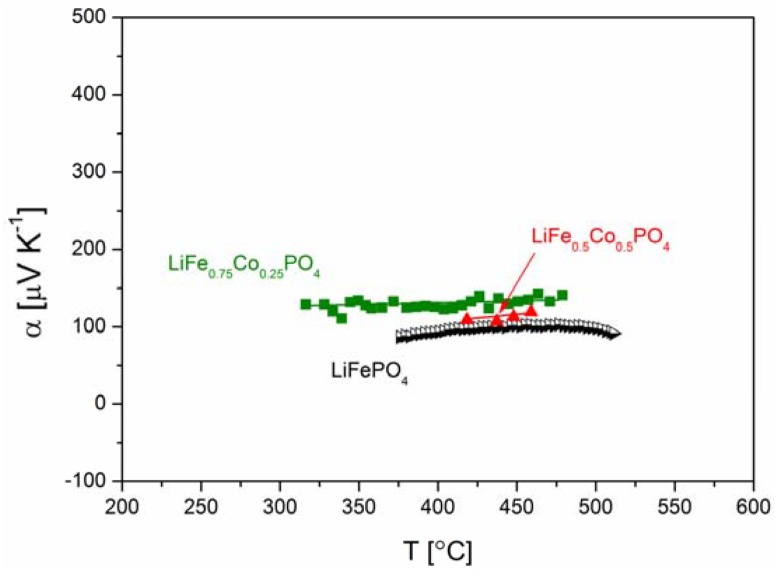
Temperature dependence of thermoelectric power of LiFe_1−*y*_Co*_y_*PO_4_.

**Figure 24:**
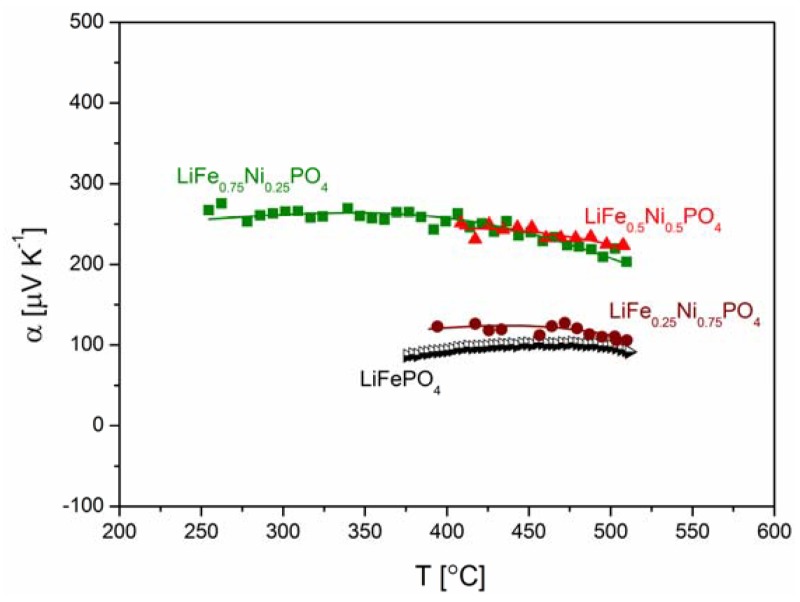
Temperature dependence of thermoelectric power of LiFe_1−*y*_Ni*_y_*PO_4_.

Figure 18, Figure 19, Figure 20, Figure 22, Figure 23 and Figure 24 show results of the measurements of electrical conductivity and thermoelectric power for phosphoolivines substituted by Mn^2+^, Co^2+^ and Ni^2+^ cations: LiFe_1−*x*_Mn*_x_*PO_4_, LiFe_1−*x*_Co*_x_*PO_4_ and LiFe_1−*x*_Ni*_x_*PO_4_ respectively. For comparison, data for pure LiFePO_4_ are also presented. Estimated values of the activation energy of electrical conductivity are collated in Figure 21. 

The obtained results of electrical conductivity for materials with 0 ≤ *y* ≤ 1 show that substitution of iron by other 3*d* metals (Mn^2+^, Co^2+^, Ni^2+^) initially causes a slight increase (half order of magnitude) of the values of σ for samples with *y* = 0.25, however, for materials with higher substitutions levels a pronounced deterioration of electrical conductivity, up to two orders of magnitude, is observed, with activation energy above 0.8 eV in some cases (Figure 21).

**Figure 25:**
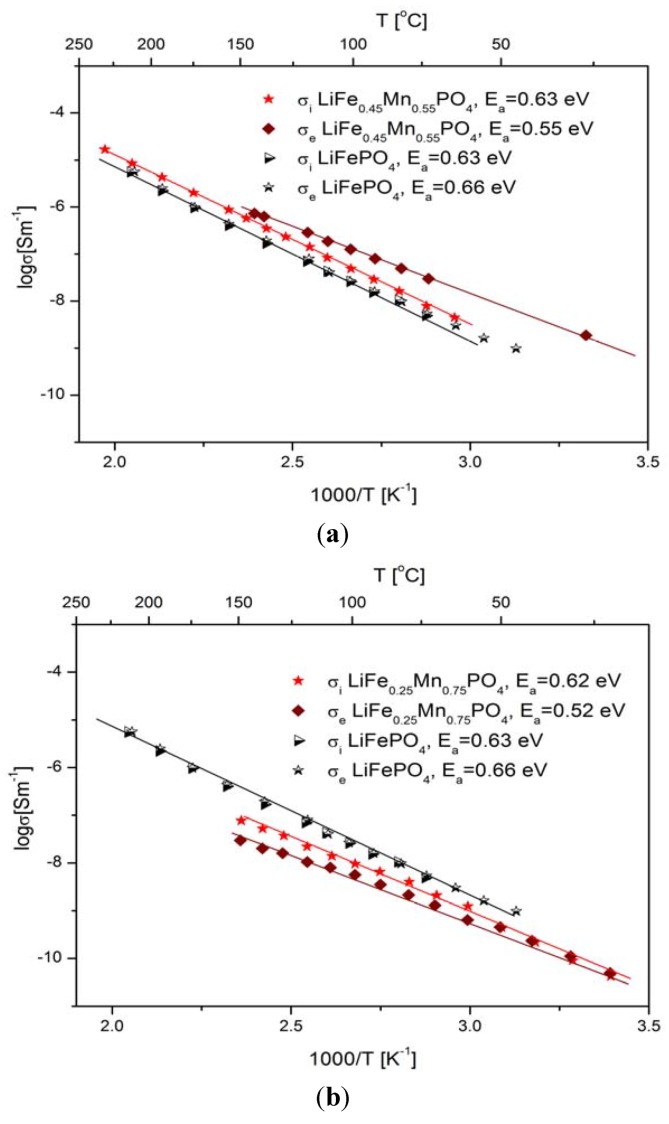
Temperature dependence of electronic and ionic component of electrical conductivity for LiFe_1−*y*_Mn*_y_*PO_4_ phosphoolivines with *y* = 0, 0.55 (**a**) and 0.75 (**b**). Data taken from [7].

Figures 25a,b show the temperature dependence of the ionic and electronic component of the electrical conductivity for phosphoolivine substituted by Mn cations. In the case of LiFe_0.45_Mn_0.55_PO_4_ compound, an increase of electronic component, of the order of one magnitude can be noticed, however, the ionic component remains almost unchanged.

**Figure 26:**
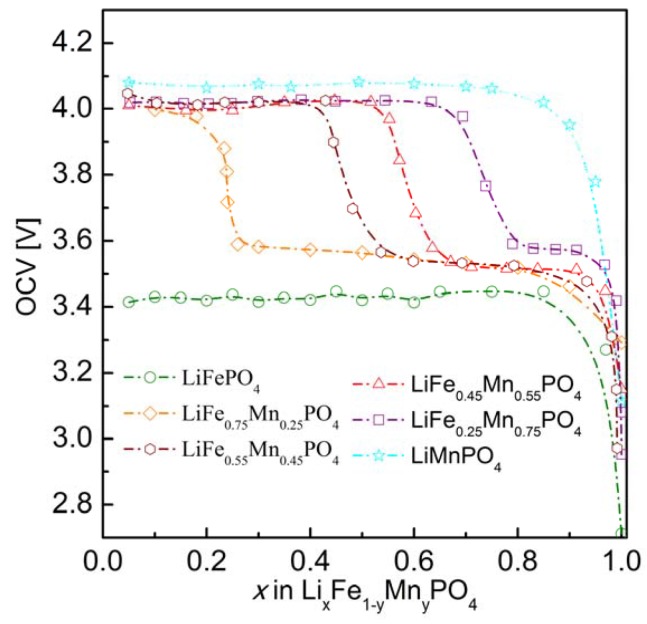
OCV curves for Li/Li^+^/Li*_x_*Fe_1−*y*_Mn*_y_*PO_4_ cells (*y* = 0, 0.25, 0.45, 0.55, 0.75 and 1) [55].

The effect of substitution of iron by other 3*d* metals (Mn^2+^, Co^2+^, Ni^2+^) on the transport properties of phosphoolivine may be analyzed by taking into account their influence on the electronic structure of the substituted materials and their electrochemical properties in Li/Li^+^/Li*_x_*Fe_1−*y*_M*_y_*PO_4_ cells. Due to a small split of energy of 3*d* orbitals in the octahedral crystal field in the phosphoolivine structure, all iron and M^2+^ metals exhibit high-spin configuration [52]. Therefore it may be written that the electronic configuration of these cations is as follows: Mn^2+^(t_g_↑)^3^(e_g_↑)^2^, Co^2+^(t_g_↑)^3^(e_g_↑)^2^(t_g_↓)^2^ and Ni^2+^(t_g_↑)^3^(e_g_↑)^2^(t_g_↓)^3^. During the electrochemical extraction of lithium from the structure (charging of the battery), in the case of 0 < *y* < 1, all substituted Li*_x_*Fe_1−*y*_M*_y_*PO_4_ phosphoolivines show a 3.5 V potential plateau related to the Fe^2+^/Fe^3+^ redox pair, as well as a second one related to the M^2+^/M^3+^ pair. For samples with *y* = 1 (end-members) the potential plateaux are equal to 4.1 V, 4.8 V and 5.1 V, respectively for Mn^2+^/Mn^3+^, Co^2+^/Co^3+^ and Ni^2+^/Ni^3+^. Exemplary data for the Li*_x_*Fe_1−*y*_Mn*_y_*PO_4_ series is shown in Figure 26. Therefore, it can be stated that in the case of initial LiFe_1−*y*_M*_y_*PO_4_ materials the Fermi level is situated in the vicinity of Fe^2+^t_g_↓ energy and the observed change of potential between the two plateaux reflects the change of energy between this level (Fe^2+^t_g_↓) and, respectively, the Mn^2+^e_g_↑, Co^2+^t_g_↓ and Ni^2+^t_g_↓ levels. It may be assumed that these electrons originating from Mn^2+^ , Co^2+^ and Ni^2+^ cations are situated deeper on the energy scale, about 1–1.5 eV deeper than the Fermi level, and therefore do not participate in the electrical conduction in the substituted samples. This in turn allows us to draw a general conclusion that in the case of the studied Fe-site substituted materials, the mechanism of magnetic polaron conduction is retained. Only the concentration of Fe^2+^t_g_↓ electrons is decreased.

According to a general equation given by Mott [53], electrical conductivity σ is correlated with concentration of polarons, in this case $c_{Fe_{Fe}^{\cdot }}$:

(1) $$\sigma =c_{Fe_{Fe}^{\cdot }}\left(1-c_{Fe_{Fe}^{\cdot }}\right)\frac{e^{2}v_{e}}{r\cdot kT}\exp\left(-2\beta \cdot r\right)\;exp\;\left(-\frac{E_{a}}{kT}\right)$$

where *β*: parameter describing distribution of wave function, *r*: distance between neighboring Fe sites, *v_e_*: frequency of hops, $c_{Fe_{Fe}^{\cdot }}$: concentration of charge carriers (holes), *e*: charge of electron, *k*: Boltzmann constant, *T*: temperature. 

Equation (2) describes the relationship between thermoelectric power α and the concentration of polarons [54]:

(2) $$\alpha =-\frac{k}{e}ln\left(\frac{c_{Fe_{Fe}^{\cdot }}}{1-c_{Fe_{Fe}^{\cdot }}}+\frac{S}{k}\right)$$

where *S*: entropy (*S*/*k* < 10 μV K^−1^)

According to Equation (1), electrical conductivity is dependent on the concentration of effective charge carriers $c_{Fe_{Fe}^{\cdot }}$, so a decrease of concentration of Fe^2+^t_g_↓ electrons, caused by the substitution of iron by 3*d* metals, deteriorates the electrical conductivity and increases the values of thermoelectric power.

#### 2.2.3. Electrochemical Properties

Figure 26 shows exemplary electrochemical OCV data for Li/Li^+^/Li*_x_*Fe_1−*y*_Mn*_y_*PO_4_ cells (*y* = 0, 0.25, 0.45, 0.55, 0.75 and 1). As mentioned in the previous section the lower-voltage plateau (≈3.5 V) corresponds to oxidation of Fe^2+^ to Fe^3+^ (removal of Fe^2+^t_g_↓ electrons), while the higher-voltage plateau (≈4 V) is related to the oxidation of Mn^2+^ to Mn^3+^ and the associated removal of Mn^2+^e_g_↑ electrons.

In order to determine the mechanism of the delithiation process in Li*_x_*Fe_1−*y*_Mn*_y_*PO_4_, structural studies were performed for materials with different concentrations of lithium (at different stages of the electrochemical delithiation process). Data were collected for the highest-conducting material with initial composition LiFe_0.45_Mn_0.55_PO_4_ (Figure 25a). Analysis of the patterns clearly shows that the observed process is a single-phase one (diffusional deintercalation-type) over the whole range of concentration 0.16 < *x*_Li_ ≤ 1. No visible reflections from the lithium-poor phase could be seen. Such a phase was evidently present in the case of electrochemical delithiation of LiFePO_4_, LiMnPO_4_ and LiFe_0.75_M_0.25_PO_4_ (M = Mn, Co and Ni). 

**Figure 27:**
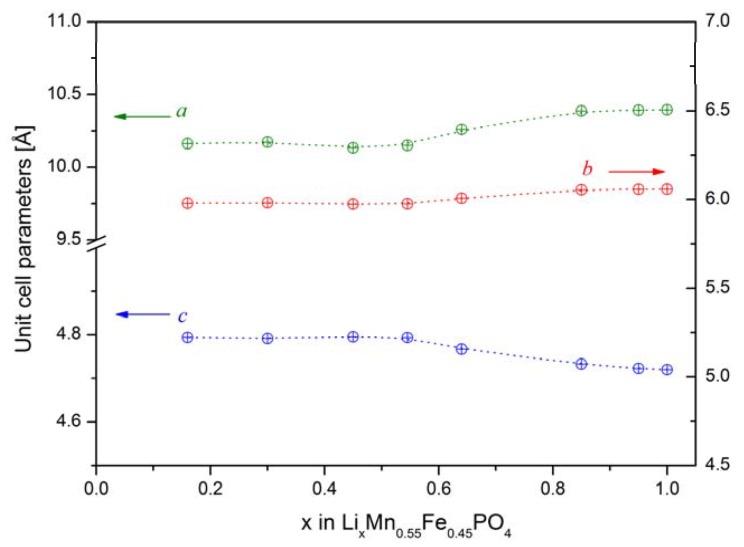
Unit cell parameters of electrochemically deintercalated Li*_x_*Fe_0.45_Mn_0.55_PO_4_ as a function of lithium content. Data taken from [7].

Figure 27 shows changes of the unit cell parameters of deintercalated Li*_x_*Fe_0.45_Mn_0.55_PO_4_ as a function of lithium content *x*, obtained from Rietveld analysis of XRD data. It can be observed that in the 0.55 ≤ *x*_Li_ < 1 range, corresponding to Fe^2+^ → Fe^3+^ oxidation, unit cell parameters vary rather strongly, in opposition to further deintercalation (*x*_Li_ < 0.55, Mn^2+^ → Mn^3+^ oxidation), for which a rather insignificant variation is observed. Structural studies presented by Yamada *et al.* [56,57,58] for Li*_x_*Fe_1−*y*_Mn*_y_*PO_4_ (0 < *y* < 1) confirm that reaction with lithium occurring at 3.5 V is a single-phase type, however these results show that at the 4 V range a change of the mechanism of electrode reaction into a two-phase type takes place. 

**Figure 28:**
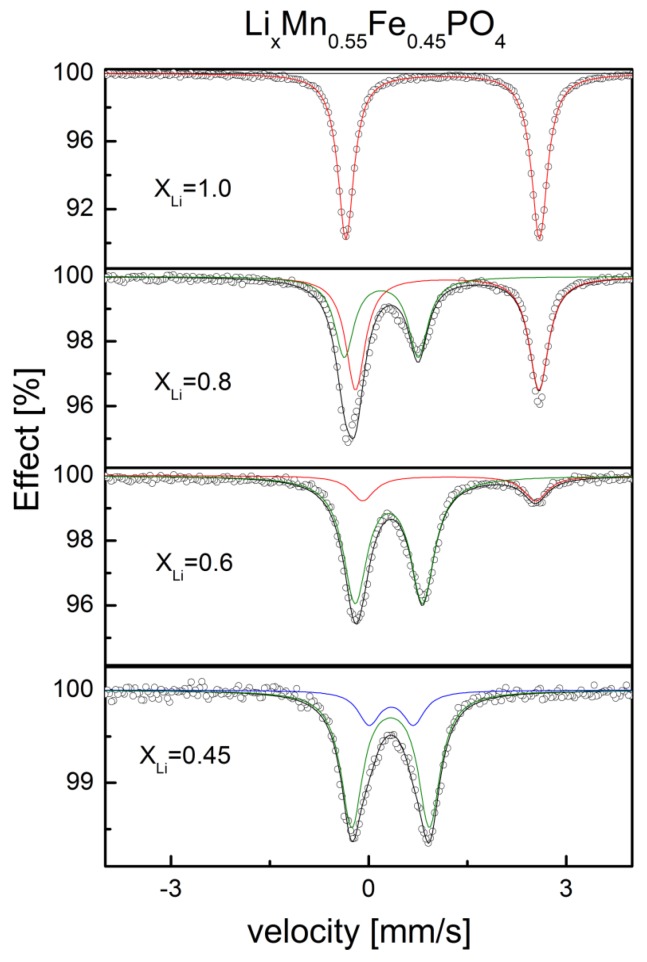
Moessbauer spectra for electrochemically deintercalated Li*_x_*Fe_0.45_Mn_0.55_PO_4_ phosphoolivines with varying lithium concentration. Data from [49].

**Figure 29:**
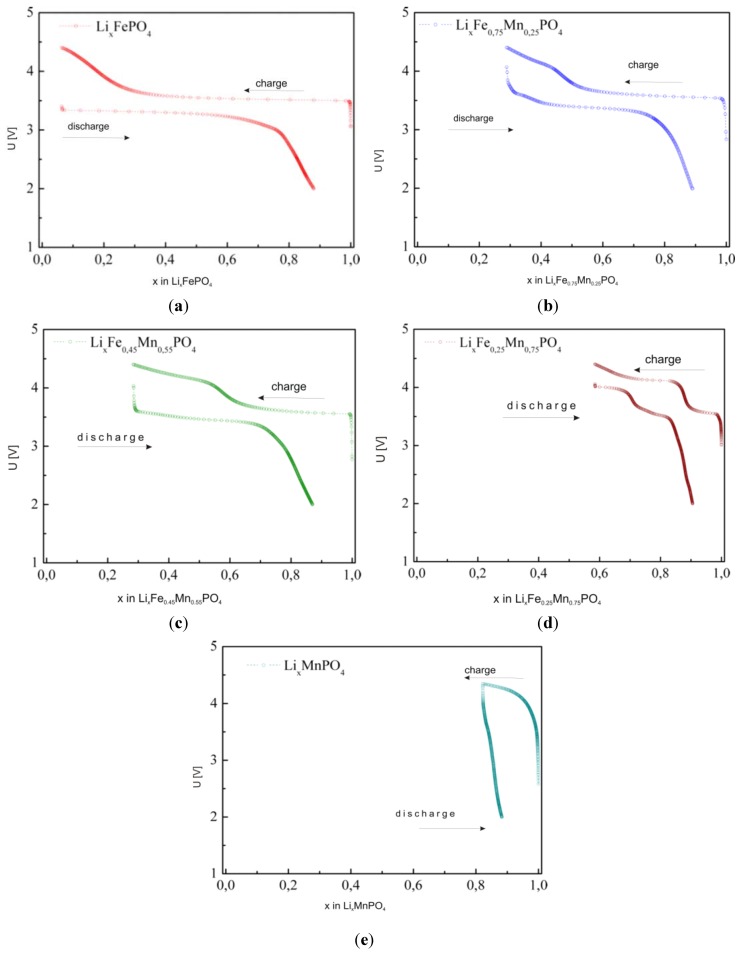
Charge/discharge curves for Li/Li^+^/Li*_x_*Fe_1−*y*_Mn*_y_*PO_4_ cells recorded with C/10 rate for materials with: (**a**) *y* = 0; (**b**) *y* = 0.25; (**c**) *y* = 0.55; (**d**) *y* = 0.75 and (**e**) *y* = 1 [55].

**Figure 30:**
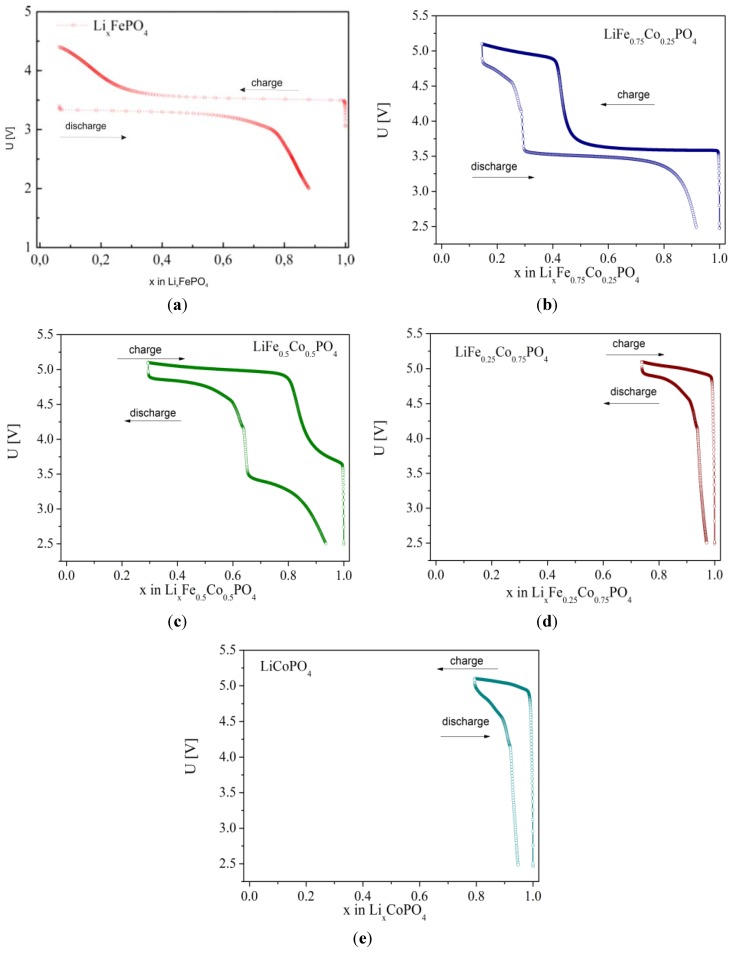
Charge/discharge curves for Li/Li^+^/Li*_x_*Fe_1−*y*_Co*_y_*PO_4_ cells recorded with C/10 speed for materials with: (**a**) *y* = 0; (**b**) *y* = 0.25; (**c**) *y* = 0.5; (**d**) *y* = 0.75 and (**e**) *y* = 1.

**Figure 31:**
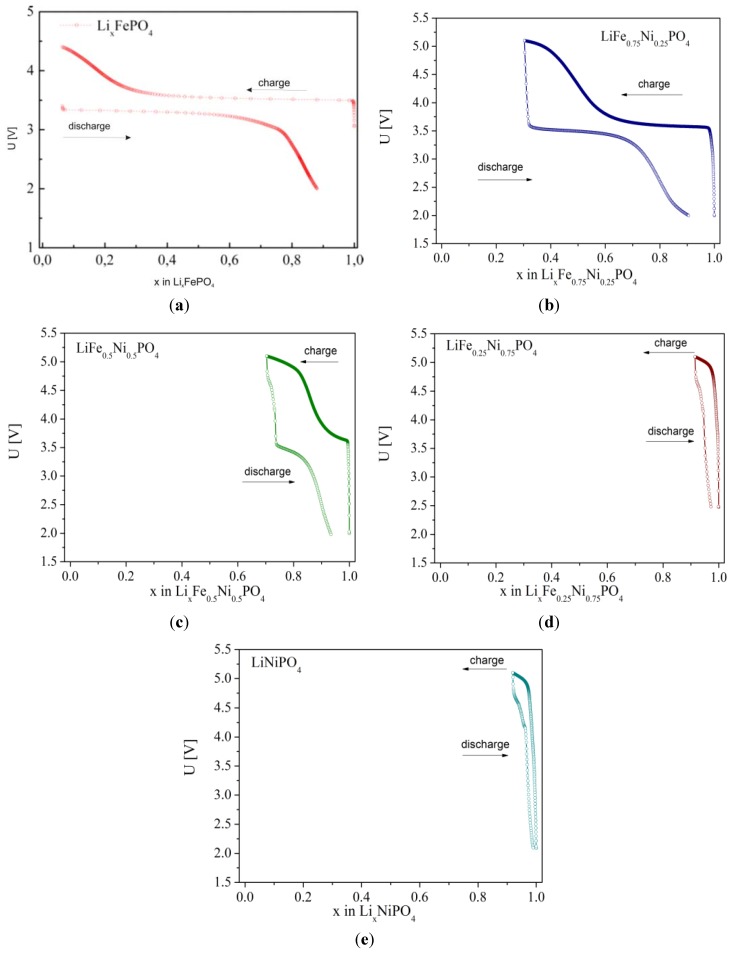
Charge/discharge curves for Li/Li^+^/Li*_x_*Fe_1−*y*_Ni*_y_*PO_4_ cells recorded with C/10 speed for materials with: (**a**) *y* = 0; (**b**) *y* = 0.25; (**c**) *y* = 0.5; (**d**) *y* = 0.75 and (**e**) *y* = 1.

Moessbauer studies of deintercalated Li*_x_*Fe_0.45_Mn_0.55_PO_4_ (Figure 28) proved that with decreasing lithium content, the Fe^2+^/Fe^3+^ ratio decreases as well. For *x*_Li_ < 0.55 in the spectra only the single charge state of iron, identified as Fe^3+^ exists, but there are two quadrupole components present, which are due to a proceeding Mn^2+^ → Mn^3+^ oxidation and the presence of different amounts of Mn^2+^ and Mn^3+^ cations in the surrounding Fe^3+^ ions. Figure 29, Figure 30 and Figure 31 show charge/discharge curves recorded for Li/Li^+^/Li*_x_*Fe_1−*y*_M*_y_*PO_4_ (M = Mn, Co, Ni) cells.

**Figure 32:**
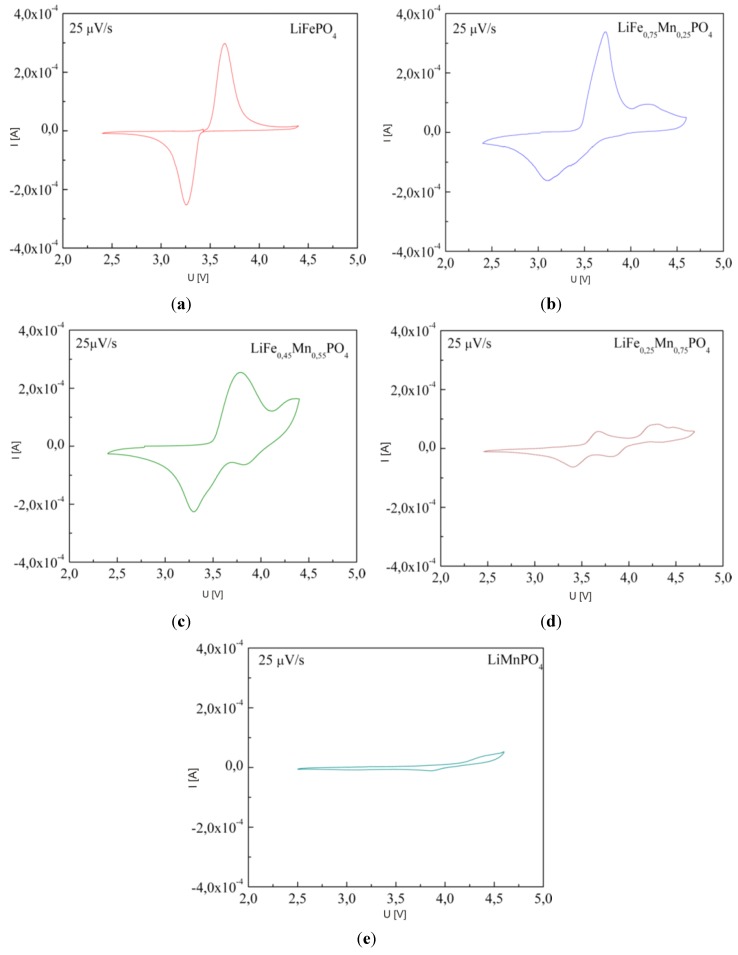
Cyclic voltammograms recorded for Li/Li^+^/Li*_x_*Fe_1−*y*_Mn*_y_*PO_4_ cells with cathode material with different concentration of manganese: (**a**) *y* = 0; (**b**) *y* = 0.25; (**c**) *y* = 0.55; (**d**) *y* = 0.75 and (**e**) *y* = 1 [55].

On analyzing charge/discharge curves, a highest reversible capacity for unsubstituted LiFePO_4_ material can be observed. Among substituted cathode materials, Li*_x_*Fe_0.75_Co_0.25_PO_4_ and Li*_x_*Fe_0.75_Mn_0.25_PO_4_ show relatively high values, respectively, 136 mAh g^−1^ and 106 mAh g^−1^. For higher substitutions, a significant deterioration of the reversibility of the cathode processes is clearly visible, which can be related to the low ionic-electronic conductivity of the materials. Additionally, the determined values of the lithium diffusion coefficient are very low, of the order of 10^−15^–10^−13^ cm^2^ s^−1^ and decrease with increasing concentration of metal M. Figure 32 shows current-voltage characteristics of Li/Li^+^/Li*_x_*Fe_1−*y*_Mn*_y_*PO_4_ cells for cathode materials with *y* = 0, 0.25, 0.45, 0.75 and 1. Two anode and cathode peaks can be observed, corresponding to oxidation/reduction of Fe^2+^/Fe^3+^ and Mn^2+^/Mn^3+^ pairs in the case of samples with 0 < *y* < 1. The highest voltage related to the Fe^2+^ → Fe^3+^ oxidation (≈3.5 V) was recorded for LiFe_0.25_Mn_0.75_PO_4_ cathode material, which is about 0.05 V higher compared to the undoped phosphoolivine. The voltage of the cathodic peak corresponding to the higher plateau, which is related to the Mn^2+^/Mn^3+^ pair, changes from 4.2 V for LiFe_0.75_Mn_0.25_PO_4_ up to 4.4 V for LiFe_0.25_Mn_0.75_PO_4_ material. With an increasing amount of substitution, the kinetics of the cathodic process worsens considerably. Evident anode and cathode peaks, which are present for LiFePO_4_, diminish and are barely visible for LiMnPO_4_. These results are in agreement with previously discussed charge-discharge curves, and are related to the deterioration of transport properties for materials with a higher concentration of metal M.

## 3. Conclusions

Based on the obtained results, the possibility of chemical modification of phosphoolivine by introduction of cation dopants in Li and Fe sublattices was evaluated. The range of solid solution formation in Li_1−3*x*_Al*_x_*FePO_4_, Li_1−4*x*_Zr*_x_*FePO_4_ and Li_1−6*x*_W*_x_*FePO_4_ materials was found to be very narrow, and consequently, transport properties of the compounds are rather weakly dependent on the chemical composition. By contrast, in the case of 3*d* metal doping in LiFe_1−*x*_M*_x_*PO_4_ (M = Mn, Co and Ni) systems, formation of solid solutions is possible in the whole composition range (0 ≤ *x* ≤ 1). Slight improvement of electrical conductivity was observed for LiFe_1−*x*_M*_x_*PO_4_ samples with *x* = 0.25. In addition, small polaron-type charge transport mechanism (hole related to Fe^3+^ (t_g_↑)^3^(e_g_↑)^2^ cation) is preserved for all substituted samples. In the case of Li*_x_*Fe_0.45_Mn_0.55_PO_4_ sample, substitution of Fe^2+^ by Mn^2+^ ions leads to the diffusional mechanism of lithium deintercalation for *x*_Li_ < 0.55. The highest reversible capacity was observed for unsubstituted LiFePO_4_, also LiFe_0.75_Co_0.25_PO_4_ and LiFe_0.75_Mn_0.25_PO_4_ showed relatively high capacity. The highest voltage related to the Fe^2+^ → Fe^3+^ oxidation (≈3.5 V) was recorded for LiFe_0.25_Mn_0.75_PO_4_ cathode material, and it is about 0.05 V higher comparing to the undoped phosphoolivine. The voltage of the cathodic peak corresponding to the Mn^2+^/Mn^3+^ redox couple, changes from 4.2 V for LiFe_0.75_Mn_0.25_PO_4_ up to 4.4 V for LiFe_0.25_Mn_0.75_PO_4_ material. For higher substitution levels, deterioration of the electrochemical performance owing to the low ionic-electronic conductivity of the materials, was detected.
